# A Heterogeneous Spiking Neural Network for Unsupervised Learning of Spatiotemporal Patterns

**DOI:** 10.3389/fnins.2020.615756

**Published:** 2021-01-14

**Authors:** Xueyuan She, Saurabh Dash, Daehyun Kim, Saibal Mukhopadhyay

**Affiliations:** Department of Electrical and Computer Engineering, Georgia Institute of Technology, Atlanta, GA, United States

**Keywords:** spiking neural network, unsupervised STDP learning, multi-objective prediction, spatiotemporal data processing, neuromorphic computing

## Abstract

This paper introduces a heterogeneous spiking neural network (H-SNN) as a novel, feedforward SNN structure capable of learning complex spatiotemporal patterns with spike-timing-dependent plasticity (STDP) based unsupervised training. Within H-SNN, hierarchical spatial and temporal patterns are constructed with convolution connections and memory pathways containing spiking neurons with different dynamics. We demonstrate analytically the formation of long and short term memory in H-SNN and distinct response functions of memory pathways. In simulation, the network is tested on visual input of moving objects to simultaneously predict for object class and motion dynamics. Results show that H-SNN achieves prediction accuracy on similar or higher level than supervised deep neural networks (DNN). Compared to SNN trained with back-propagation, H-SNN effectively utilizes STDP to learn spatiotemporal patterns that have better generalizability to unknown motion and/or object classes encountered during inference. In addition, the improved performance is achieved with 6x fewer parameters than complex DNNs, showing H-SNN as an efficient approach for applications with constrained computation resources.

## 1. Introduction

Spiking neural network (SNN) (Maass, 1997; Gerstner and Kistler, 2002b; Pfeiffer and Pfeil, 2018) is a dynamical system with bio-inspired neurons (Izhikevich, 2003) and learning behaviors (Dan and Poo, 2006; Caporale and Dan, 2008). In rate-encoded SNN, inputs are represented as spike trains with varying (pixel dependent) frequency and patterns within input spikes can be learned without supervision using spike-timing-dependent plasticity (STDP) (Bell et al., 1997; Magee and Johnston, 1997; Gerstner and Kistler, 2002a). The event-driven nature of SNN operation also promises high energy-efficiency during real-time inference (Roy et al., 2019). Over the years, SNN has shown success in spatial processing such as image classification. Although, many large scale spatial SNNs depend on conversion from DNN (Diehl et al., 2015; Rueckauer et al., 2017; Sengupta et al., 2019) or supervised training (Lee et al., 2016; Nicola and Clopath, 2017; Huh and Sejnowski, 2018), more recently, unsupervised learning has shown promising results (Lee et al., 2018; Srinivasan and Roy, 2019). However, SNNs for processing temporal or spatiotemporal data are still primarily based on recurrent connections (DePasquale et al., 2016; Bellec et al., 2018) and use supervised training (Stromatias et al., 2017; Wu et al., 2018), leading to high network complexity for processing spatiotemporal data and high demand for labeled data. Recently, SNN has also been explored for optical flow applications, such as Spike-FlowNet (Lee et al., 2020), which is based on self-supervision; with STDP-based learning, it is possible to implement SNN for optical flow (Paredes-Vallés et al., 2020), but learning is limited to only short-term temporal patterns.

In this paper, we present a novel heterogeneous SNN (H-SNN) architecture that exploits inherent dynamics of spiking neurons for unsupervised learning of complex spatiotemporal patterns. Our key innovation is to use feedforward connections of spiking neurons with different dynamics to represent memory of different time-scales and learn temporal patterns. This eliminates the need for recurrent connection present in state-of-the-art SNNs used for temporal learning (DePasquale et al., 2016; Bellec et al., 2018; Wu et al., 2018; Lee et al., 2020). Moreover, we adapt spiking convolution modules (Kheradpisheh et al., 2018) to the network architecture. As a result, H-SNN is able to extract distinguishing temporal and spatial features from spatiotemporal inputs using only STDP based unsupervised training. To the best of our knowledge, H-SNN is the first STDP-learned multi-objective SNN for predicting object class and motion. More specifically, the following key contributions are made:

- We propose a novel spiking network architecture and STDP based unsupervised learning process for predicting object class and motion dynamic from spatiotemporal inputs.
- We design neurons with different dynamics and network layers with crossover connections to hierarchically form short and long term memory used to learn complex temporal patterns.
- We present spiking convolutional network with local/cross-depth inhibition to learn motion invariant spatial features from augmented dataset.
- We analytically show that heterogeneous neuronal dynamics and multi-path connection can represent distinguishable features of temporal patterns without any recurrent connection.

**Figure 1:**
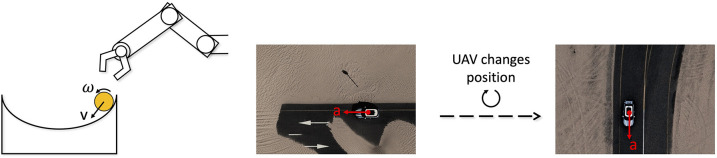
Objects with mixed dynamics in computer vision applications: robots interacting with a rolling ball in a convex **(Left)**; an UAV analyzing movement of a vehicle **(Right)**.

The effectiveness of H-SNN is demonstrated with computer vision tasks involving dynamic data set, considering that many neural network applications involve perception of objects in motion (Marković et al., 2014; Baca et al., 2018). Figure 1 illustrates examples of such applications, where a robotic arm is interacting with a rolling object, and where an unmanned aerial vehicle (UAV) is analyzing a moving car. The object motion can be a mixture of translation and rotation, both with constant or changing speed. A neural network is required to perform two tasks: identify class of the object and understand dynamic of the motion. In real world implementations where unforeseen conditions might be encountered, the neural network must also be able to correctly classify known objects with unseen motion and recognize known motion of unknown objects. Thus, for networks that rely on camera as input, the constant transformation of pixel-level information makes the preceding problem challenging.

In our experiment, we first test the networks on a human gesture dataset captured by event camera (Amir et al., 2017). Datasets consist of frame sequences with various linear (translation) and angular (rotation) motions created from an aerial image dataset (Xia et al., 2017) and Fashion-MNIST (Xiao et al., 2017) are also tested for multi-objective prediction. We compare H-SNN to deep learning approaches including 3D Convolutional Neural Network (CNN) and CNN combined with recurrent neural network (RNN), as well as SNN trained with back-propagation. Results show that H-SNN has comparable (or better) prediction accuracy than baselines while having similar or less network parameters.

## 2. Materials and Methods

### 2.1. Deep Learning for Learning Spatiotemporal Patterns

The spatiotemporal input data can be processed using deep neural network (DNN) with 3D convolution kernel (Tran et al., 2015) or recurrent connection (Donahue et al., 2015). However, in those spatiotemporal networks, transformation invariance is not explicitly imposed. To achieve transformation equivalent feature extraction, several DNN architectures have been proposed (Cheng et al., 2016; Weiler et al., 2018; Zhang, 2019). While it is possible to apply those designs in the spatiotemporal network, it has been demonstrated that DNNs are invariant only to images with very similar transformation as that in the training set (Azulay and Weiss, 2018), which indicates that DNNs generalize poorly when learning feature transformations. As a result, an increased amount of network parameters are required for conventional DNN to achieve transformation invariant classification. For example, in Weiler et al. (2018), each pre-defined rotation angle requires an additional set of CNN filters; in data augmentation based approaches such as Cheng et al. (2016), more parameters are needed to learn all the rotated features. Together with the combination of 3D kernel or recurrent connections, this leads to large complexity for spatiotemporal networks.

### 2.2. Spiking Neuron and Synapses

Spiking neural network uses biologically plausible neuron and synapse models that can exploit temporal relationship between spiking events (Moreno-Bote and Drugowitsch, 2015; Lansdell and Kording, 2019). There are different models that are developed to capture the firing pattern of real biological neurons. We choose to use Leaky Integrate-and-Fire (LIF) model in this work described by:

(1) $$\begin{array}{l} \tau_{m}\frac{dv}{dt}=a+R_{m}I-v\;\text{and }v=v_{reset},\text{if }v>v_{threshold} \end{array}$$

Here, *R*_*m*_ is membrane resistance and τ_*m*_ = *R*_*m*_*C*_*m*_ is the time constant with *C*_*m*_ being membrane capacitance. *a* is a parameter used to adjust neuron behavior during simulation. *I* is the sum of current from all synapses that connects to the neuron. A spike is generated when membrane potential *v* cross threshold and the neuron enters refractory period, during which the neuron can not spike again.

In SNN, two neurons connected by one synapse are referred to as pre-synaptic neuron and post-synaptic neuron. Conductance of the synapse determines how strongly two neurons are connected and learning can be achieved through modulating the conductance following an algorithm named spike-timing-dependent-plasticity (STDP) (Gerstner et al., 1993; Gerstner and Kistler, 2002a), which has been applied in SNNs designed for computer vision applications (Masquelier and Thorpe, 2007; Diehl and Cook, 2015). There are two types of synaptic weight modulation in STDP: long-term potentiation (LTP) and long-term depression (LTD). More specifically, LTP is triggered when post-synaptic neuron spikes closely after a pre-synaptic neuron spike, indicating a causal relationship between the two events. On the other hand, when a post-synaptic neuron spikes before pre-synaptic spike arrives or without receiving a pre-synaptic spike at all, the synapse goes through LTD. There is an exponential relationship between the time difference of spikes Δ*t* (Bi and Poo, 2001) and the current level of conductance (Querlioz et al., 2013). In this work, the magnitude of LTP and LTD is determined as follows:

(2) $$\begin{array}{l} \Delta G_{p}=\alpha_{p}e^{-\Delta t\left(G-G_{min}\right)/\left(\tau_{pot}\left(G_{max}-G_{min}\right)\right)} \end{array}$$

(3) $$\begin{array}{l} \Delta G_{d}=\alpha_{d}e^{-\Delta t\left(G_{max}-G\right)/\left(\tau_{dep}\left(G_{max}-G_{min}\right)\right)} \end{array}$$

In the functions above, Δ*G*_*p*_ is the magnitude of LTP actions, and Δ*G*_*d*_ is the magnitude of LTD actions. α_*p*_, α_*d*_, *G*_*max*_, and *G*_*min*_ are parameters that are tuned based on specific network configurations. τ_*dep*_ and τ_*pot*_ are time constant parameters. Δ*t* is determined by subtracting the arrival time of the pre-synapse spike from that of the post-synapse spike (*t*_*post*_ − *t*_*pre*_).

### 2.3. The Proposed Method

#### 2.3.1. Heterogeneous Neuron Dynamics

Prior SNN designs mostly focus on using neurons with the same dynamics through the network. In contrast, H-SNN combines neurons with different dynamics to facilitate learning and memory of different length separately in a heterogeneous network. As neurons are used as the basic element of information retention, long and short retention length can be achieved if neurons have different decay rates. For simplicity, let $b=-\frac{1}{\tau_{m}}$ and $c=\frac{R_{m}}{\tau_{m}}$. Consider a spiking neuron that receives input current *I* at *t* = 0, time for its membrane potential to decay to *v*_*reset*_ is:

(4) $$\begin{array}{l} t_{decay}=\frac{1}{b}ln\left(v_{reset}+\frac{a}{b}\right)-\frac{1}{b}ln\left(v_{reset}+\frac{a}{b}+cI\right) \end{array}$$

Equation (4) suggests that, by adjusting the parameters *a*, *b* and *c* in Equation (1), different information retention period can be achieved. Particularly, in H-SNN, three types of spiking neurons are used:

- **Learner neuron**, which has a balanced decay rate and input response designed to optimize STDP learning, is similar to neurons used in previous works (Querlioz et al., 2013; Lee et al., 2018; Srinivasan and Roy, 2019). Its parameters are referred to as {*a*_*ln*_, *b*_*ln*_, *c*_*ln*_}.
- **Short-term neuron**, with parameters {*a*_*stn*_, *b*_*stn*_, *c*_*stn*_}, has *a*_*stn*_ < *a*_*ln*_ and *b*_*stn*_ > *b*_*ln*_ to create higher decay rate. It is used to extract short term patterns from input features.
- **Long-term neuron**, with parameters {*a*_*ltn*_, *b*_*ltn*_, *c*_*ltn*_}, has lower decay rate than learner neuron, as *a*_*ltn*_ > *a*_*ln*_ and *b*_*ltn*_ < *b*_*ln*_. It is designed for long term pattern recognition.

**Figure 2:**
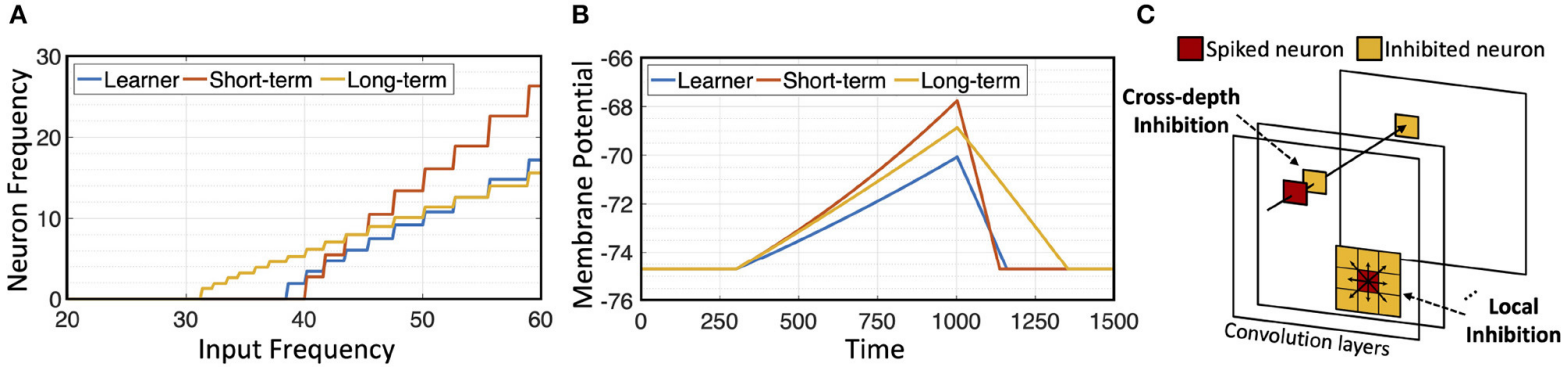
Neurons and inhibition: **(A)** Neuron response to input frequency; **(B)** neuron decay rate; **(C)** illustration of cross-depth and local inhibition.

Since membrane potential of long term memory neuron decays slower, under the same input signal, it can potentially produce spike frequency that dominates the short term memory neuron when the two are placed in parallel. To prevent this, *c*_*stn*_ > *c*_*ltn*_ is used. Figure 2A shows responses of different neurons to pre-synaptic frequency. Long-term neuron is able to respond to lower frequency input, due to its lower decay rate, but its post-synaptic frequency increases slowly compared to the short-term neuron. Figure 2B shows that short-term and long-term neurons gain membrane potential faster than learner neuron with a given input current, but they also exhibit different decay rates when input current is zero.

#### 2.3.2. Network Architecture

**Figure 3:**
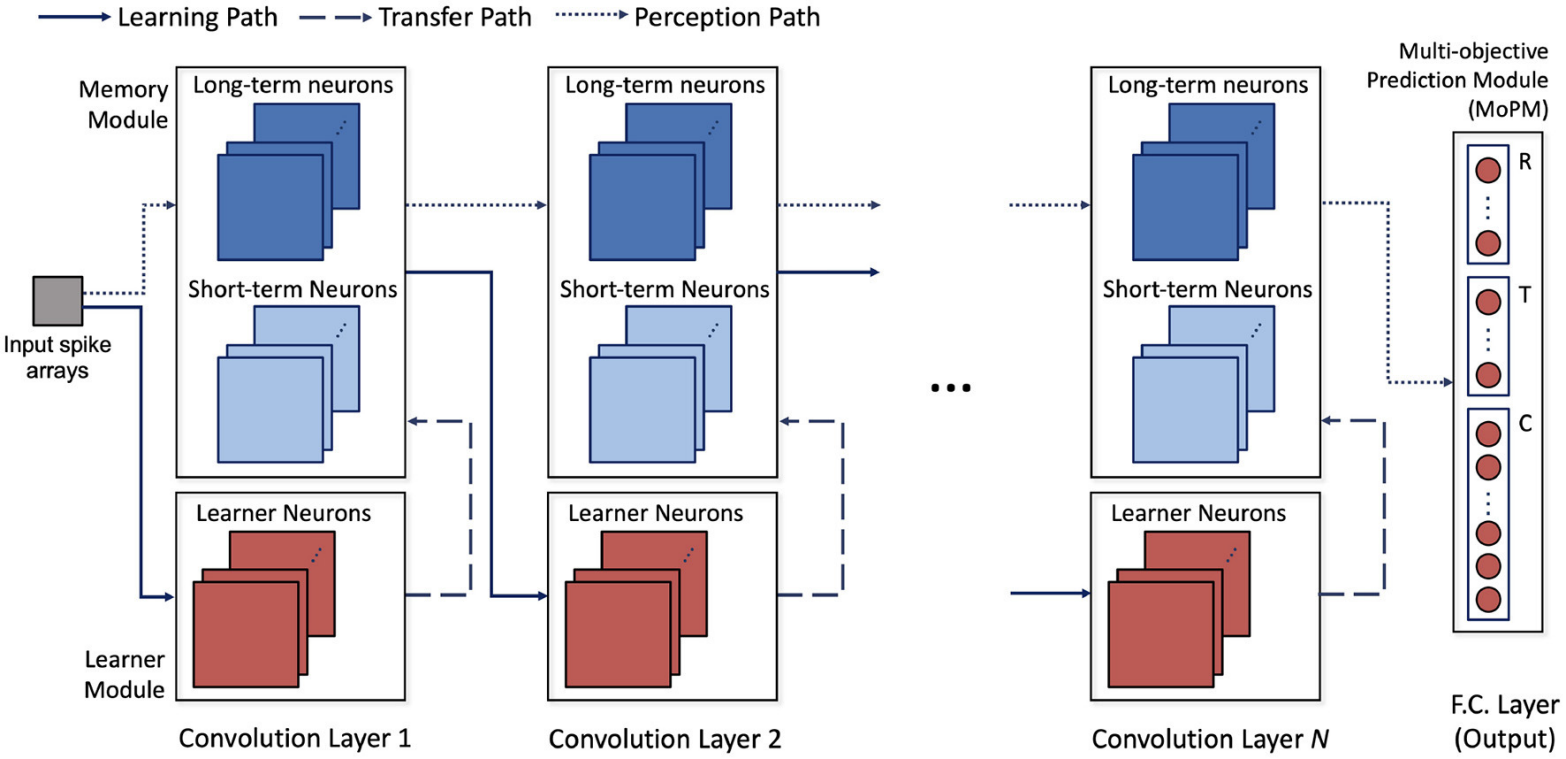
Architecture of the proposed network; network operation involves data flow of three paths: learning path where STDP learning is used to modulate synapse conductance, transfer path where learned features are transferred to long and short term neurons, and perception path, where perceived input spatiotemporal features are encoded in spikes.

The architecture of H-SNN is shown in Figure 3. Three types of modules are connected by three types of data flow paths between network layers. Spike signal from each layer's memory module is sent through perception path to deeper layers. Learning path connects memory module to learner module in the next layer to enable STDP learning. The learned synapse conductance is transferred from learner module to memory module in the same layer. Modules are built with neurons of specific dynamics as mentioned before. Each convolution layer contains a learner module and a memory module. Two inhibition schemes are implemented within the convolutional layers: cross-depth, and local (Figure 2C). Cross-depth inhibition is implemented to create competition between neurons with the same receptive field. This prevents more than one kernel from learning the same pattern. Local inhibition, where the spike of one neuron inhibits surrounding neurons in the same depth, is used to help the network to better detect and learn translation invariant features.

Learner module is responsible for facilitating STDP learning. All the spiking neurons in this module are learner neurons, and local inhibition is combined with cross-depth inhibition. For memory module, a combination of long-term and short-term neurons are used. Synapses in memory module are used only for perception thus not modified by STDP learning. Cross-depth inhibition is not implemented to accelerate neuron response and mitigate the diminishing spike frequency issue. The last layer is a multi-objective prediction module (MoPM) with each neuron fully connected to the previous layer. As will be discussed later, MoPM is fine-tuned with supervision. To facilitate the common conversion process, MoPM consists of all standard learner neurons. Inside the MoPM, neurons are indexed as *N*_*i,j*_ where *i* represents one objective (section) and each *j* represent one label (class) within that section. Here, section-lateral inhibition is implemented, which means that spiking of neuron *N*_*i,j*_ sends inhibition signal to all neurons in section *i* while other sections are unaffected. This enables H-SNN to simultaneously generate prediction for independent objectives.

#### 2.3.3. Learning Process

For each input sequence, all frames are converted to a 2D array of spike train; the frequency of spike train for a pixel is proportional to the pixel's intensity (rate encoding). The network receives one frame at a time and observes it for a period (*t*_*train*_ chosen based on input frequency range) to generate sufficient spiking events. After all frames are learned the process repeats for the next sequence.

The learning of the network proceeds layer-wise. During learning of layer 1, neurons in the learner module receive input spikes and perform STDP learning. The learned conductance matrix is transferred to long-term and short-term neurons. Next, learning of layer 2 begins. In this step, memory neurons in layer 1 receive input spikes and generate spikes for the learner neurons in layer 2. After the learner neurons complete learning of all training sequences, the conductance matrix is transferred to layer 2 memory neurons. This process repeats for all convolution layers. While in one layer, the learner neurons exhibit different neuron activity with the long-term and short-term neurons, such layer-wise learning process allows the next layer to learn the changed spiking pattern using STDP. Since multi-layer SNN experiences diminishing spiking frequency along network layers, threshold of neurons in the memory module are scaled down to produce higher output spiking frequency. The scaling factor for each layer is tuned as network hyperparameter, and its value is kept uniform within one layer to prevent distortion of output pattern. During training of the final fully connected layer (MoPM), the perception path produces spikes for each training sequence and spike frequency of all memory neurons in convolution layer *n* is calculated. The last layer is trained with a conventional stochastic gradient descent method to predict multiple objectives. The objective function is to minimize binary cross entropy loss between label vectors that are one-hot encoded for each prediction target and the layer output.

#### 2.3.4. Memory Pathway - Hierarchical Memory Formation in H-SNN

**Figure 4:**
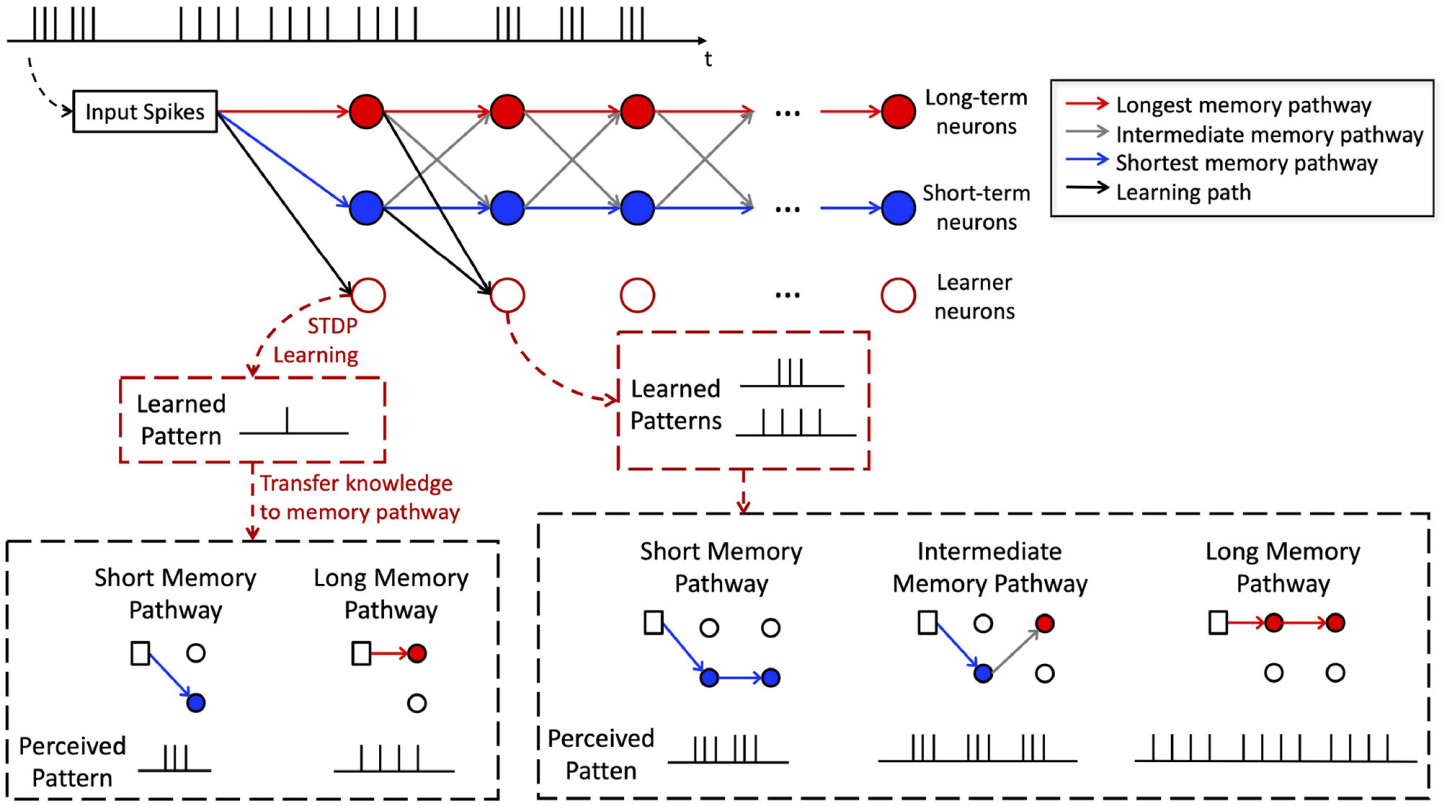
Illustration of memory pathway and hierarchical learning in H-SNN.

Within the perception path, long-term and short-term neurons in different layers are connected with crossover connections. This establishes different memory pathways as shown by the red, gray and blue lines in Figure 4. More specifically, we define a memory pathway as one trace of stacked connection of long-term and short-term neurons from the first memory module to the last.The memory pathways in H-SNN have a wide range of time scales. Connections consist of entirely short-term neurons, long-term neurons, and mixture of both types of neurons create memory pathways of shortest, longest, and intermediate time scales, respectively.

To better illustrate this, an example is shown in Figure 4. The memory pathways enable hierarchical learning of temporal patterns for the target spike train shown at the top of the figure. Learner neurons in the first layer extract correlation information from the immediate past (single spike in Figure 4) and transfer such knowledge to neurons in memory pathway (two different compositions of a single spike in Figure 4). The memory of layer 1 creates a higher level of temporal abstraction of input features that are transmitted as spike inputs to the learner neurons in layer 2. Hence, learner neuron in layer 2 learns compositions of the higher level temporal patterns perceived by memory modules in layer 1 (see bottom of Figure 4 for illustration). This process is repeated throughout the learning process creating a hierarchical learning of temporal features with learner neurons in the last layer learning highest level temporal features over the longest time-window. In other words, the equivalent STDP learning window expands along network layers, with long memory pathways creating the faster expansion and short memory pathways creating the slower expansion. Temporal features of different scales can therefore be learned without supervision.

### 2.4. Spatiotemporal Pattern Representation in H-SNN

For H-SNN to predict object class and motion dynamic, memory modules needs to transform the input sequences to output space that contains spatial (motion invariant) features of target classes and spatiotemporal features of target dynamics. The last layer of H-SNN trained with SGD can statistically correlate those attributes in the reduced dimension to output targets. As H-SNN uses spatial architecture based on spiking convolutional module, H-SNN models spatial features similar to a traditional CNN. However, unlike the global optimization of parameters using gradient descent in CNN, H-SNN uses local, unsupervised STDP to learn spatial patterns in a layer-by-layer fashion, such as shown in prior works (Kheradpisheh et al., 2018; Srinivasan and Roy, 2019).

A network must memorize distinguishable information over various time scales to model temporal patterns. Since H-SNN does not use recurrent connections, we analytically show that the feed-forward connections of spiking neurons with different dynamics can represent various temporal patterns, through proving two properties of H-SNN: output of different memory pathways is distinguishable and different memory pathways can retain temporal information of different length.

We first show that memory pathways have different response function, i.e. different mapping from input spike pattern to output spike pattern (Lemma 2.1). As information is represented by spike frequency (rate encoding), this proves that each memory pathway has distinguishable information (Lemma 2.2). Next, we demonstrate that different memory pathways represent information over different time scales (Lemma 2.4). The following steps show that the H-SNN can represent temporal patterns without using recurrent connections.

#### 2.4.1. Neuron Decay Rate

First, consider the LIF neuron as described by (1), as reproduced here with parameters *b* and *c*:

$$dv/dt=a+bv+cI\;\text{and }v=v_{reset},\;\text{if }v>v_{threshold}$$

Without input current, the differential equation for membrane potential of neuron *m* can be re-written a first order separable ordinary differential equation, and its solution leads to:

(5) $$\begin{array}{c} \frac{1}{a+bv_{m}}dv_{m}/dt=1\\ v_{m}\left(t\right)=\frac{1}{b}e^{bt+Cb}-\frac{a}{b} \end{array}$$

Here, C is the constant of integration. A single input spike with current *I* drives membrane potential to:

$$v_{m}^{ltn}=v_{reset}+cI$$

Consider initial condition at *t* = 0 with *v*_*m*_ = *v*_*reset*_ + *cI*, value of integration constant *C* can be found, and by substituting it (5) can be rewritten:

$$\begin{array}{c} C=\frac{1}{b}ln\left(bv_{reset}+bcI+ab\right)\\ v_{m}^{ltn}\left(t\right)=\left(v_{reset}+cI+\frac{a}{b}\right)e^{bt}-\frac{a}{b} \end{array}$$

After neuron *m* received the input spike, the time it takes for the membrane potential to decay to $v_{m}^{ltn}=v_{reset}$ is:

$$t_{decay}=\frac{1}{b}ln\left(v_{reset}+\frac{a}{b}\right)-\frac{1}{b}ln\left(v_{reset}+\frac{a}{b}+cI\right)$$

It can be observed that decay rate is different for the three types of neuron and their parameters {*a, b, c*} differ.

#### 2.4.2. Distinguishability of Memory Pathways

We first derive the spike response function of memory pathways, which formulates output spike frequency given input spike frequency. Based on this, we show the distinguishability of memory pathways by analyzing their response functions.

**Lemma 2.1**. *Let {n_1_, n_2_, ...n_m_} be a list of sequential connected spiking neurons that have uniform refractory period r, and minimal number of input spike needed to reach spiking threshold {γ_1_, γ_2_, ...γ_m_}. With f_in_ being the input spike frequency to n_1_, the output spike frequency of n_m_ is*:

$$f_{n_{m}}={\left(r+\frac{\prod_{i=1}^{m}\gamma_{i}}{f_{in}}+r\sum_{k=0}^{m-2}\prod_{j=0}^{k}\gamma_{m-j}\right)}^{-1}$$

*Proof*. Spiking event at time *t*^*i*^ can be marked with Dirac delta function δ(*t* − *t*^*i*^). For a spiking neuron *m*, input current from all pre-synaptic neurons is $I_{m}=\underset{n,i}{\sum }G_{mn}\delta \left(t-t_{n}^{i}\right)$, where *G*_*nm*_ is the synapse conductance between neuron *n* and *m*, and the sum is over all pre-synaptic spiking events. Integration of the input current can be performed for each pre-synaptic spike. With initial state at *t* = 0 with *v* = *v*_*reset*_, solving (1) leads to:

(6) $$\begin{array}{l} v_{m}\left(t\right)=v_{reset}e^{bt}-\frac{a}{b}\left(1-e^{bt}\right)+ce^{bt}\underset{n}{\sum }\underset{i}{\sum }G_{mn}\int_{0}^{t}\delta \left(t-t_{n}^{i}\right)e^{-bt}dt \end{array}$$

Now consider neuron *m* with one pre-synaptic neuron that has output frequency *f*_*in*_ and *t*^0^ = 0, the time *t*^γ^ for neuron *m* to reach spiking state is $v_{m}\left(t^{\gamma }\right)>=v_{th}$, where *v*_*th*_ is the threshold voltage. Since the membrane potential increases at time of *t*^*i*^ and decays otherwise, *t*^γ^ will be one instance of *t*^*i*^. When we set *t* = *t*^*i*^ and expand the equation for *v*_*m*_, we have:

(7) $$\begin{array}{l} v_{m}\left(t^{i}\right)=\left(v_{reset}+\frac{a}{b}\right)e^{bt^{i}}-\frac{a}{b}+cGe^{bt^{i}-bt^{1}}+cGe^{bt^{i}-bt^{2}}+...+cG \end{array}$$

The post-synaptic neuron *m* has output spike frequency that can be found with:

(8) $$\begin{array}{l} f_{out}=F\left(f_{in}\right)=\frac{1}{T+r}=\frac{1}{\left(\gamma /f_{in}\right)+r}=\frac{f_{in}}{\gamma +rf_{in}} \end{array}$$

where *r* is the refractory period, and $t^{\gamma }=\left\{\min t^{\gamma }|v_{m}\left(t^{\gamma }\right)\geq v_{th}\right\}$. *F*(*f*_*in*_) is referred to as the neuron response function. Consider a long-term neuron and a short-term neuron connected in series with one input neuron at spiking frequency *f*_*in*_, the response function would be a composition of two neurons' response function:

(9) $$\begin{array}{l} \left(F_{stn}◦F_{ltn}\right)\left(f_{in}\right)={\left(\frac{\gamma_{ltn}\gamma_{stn}}{f_{in}}+\gamma_{stn}r+r\right)}^{-1} \end{array}$$

By doing the same composition for more neurons, we can generalize the pattern, and response function of memory pathway (Φ(*f*_*in*_)) that consists of *m* neurons can be derived as follows:

(10) $$f_{n_{m}}=\Phi (f_{in})={\left(r+\frac{\prod_{i=1}^{m}\gamma_{i}}{f_{in}}+r\sum_{k=0}^{m-2}\prod_{j=0}^{k}\gamma_{m-j}\right)}^{-1}$$

The proof is complete.

**Lemma 2.2**. *The response function (10) for different memory pathways are distinct from each other*.

*Proof*. Consider memory pathway formed by two neurons *p*_1_ = {*n*_*ltn*_, *n*_*stn*_} with γ_*ltn*_, γ_*stn*_ connected in order, and another memory pathway formed by *p*_2_ = {*n*_*stn*_, *n*_*ltn*_} with γ_*stn*_, γ_*ltn*_ connected in order. The response function of the two memory pathways is:

(11) $$\begin{array}{l} \Phi_{p_{1}}\left(f_{in}\right)={\left(\frac{\gamma_{ltn}\gamma_{stn}}{f_{in}}+\gamma_{stn}r+r\right)}^{-1}\;\text{and}\\ \Phi_{p_{2}}\left(f_{in}\right)={\left(\frac{\gamma_{stn}\gamma_{ltn}}{f_{in}}+\gamma_{ltn}r+r\right)}^{-1} \end{array}$$

It can be observed that Φ_*p*_1__ ≠ Φ_*p*_2__, and that response function (10) is dependent on the order of γ. Each memory pathway in H-SNN has sequentially connected neurons that form an ordered list of γ. Since all memory pathways have different connection sequences, which leads to different sequences of γ, response function Φ is different for each memory pathway. The proof is complete.

#### 2.4.3. Retention Length of Memory Pathways

Here, we first prove the existence of cut-off frequency of spiking neurons, followed by analysis of retention length of memory pathways.

**Lemma 2.3**. *There exists a cut-off pre-synaptic spike frequency to a post-synaptic neuron below which the post-synaptic neuron cannot spike*.

*Proof*. From the membrane potential equation derived in the main paper for Lemma 3.1:

$$v_{m}\left(t^{i}\right)=\left(v_{reset}+\frac{a}{b}\right)e^{bt^{i}}-\frac{a}{b}+cGe^{bt^{i}-bt^{1}}+cGe^{bt^{i}-bt^{2}}+\ldots +cG$$

Since $\Delta t=\frac{1}{f}=t^{i+1}-t^{i}$, subtracting membrane potential values at two consecutive *t*_*i*_ provides:

(12) $$\begin{array}{l} \Delta v_{m}=v_{m}\left(t^{i+1}\right)-v_{m}\left(t^{i}\right)=\left(v_{reset}+\frac{a}{b}\right)e^{bt^{i+1}}-\left(v_{reset}+\frac{a}{b}\right)e^{bt^{i}}\\ \;+cGe^{bt^{i+1}-bt^{1}} \end{array}$$

setting *t*^1^ to zero, we have:

(13) $$\begin{array}{l} \Delta v_{m}=\left(v_{reset}+\frac{a}{b}\right)e^{bt^{i}}\left(e^{b\Delta t}-1\right)+cGe^{bt^{i}}e^{b\Delta t}\\ \Delta v_{m}=\left(\left(v_{reset}+\frac{a}{b}\right)\left(e^{b\Delta t}-1\right)+cGe^{b\Delta t}\right)e^{bt^{i}} \end{array}$$

As the term $\left(\left(v_{reset}+\frac{a}{b}\right)\left(e^{b\Delta t}-1\right)+cGe^{b\Delta t}\right)$ does not depend on *t*^*i*^, *v*_*m*_ is either strictly increasing, staying the same or decreasing with higher *t*^*i*^. This indicates that, when Δ*v*_*m*_ ≤ 0 the post-synaptic neuron can never spike regardless of how many pre-synaptic spike it receives. For post-synaptic neuron with specific parameters and synapse conductance *G*, $\left(\left(v_{reset}+\frac{a}{b}\right)\left(e^{b\Delta t}-1\right)+cGe^{b\Delta t}\right)$ depends on Δ*t*. Since $\Delta t=\frac{1}{f}$, there exists a value of pre-synaptic frequency *f* below which the post-synaptic neuron cannot spike. The proof is complete.

**Lemma 2.4**. *The duration for which each memory pathway can store information is different*.

*Proof*. Given a memory pathway consist of neurons {*n*_1_, *n*_2_, ...*n*_*m*_} connected in order with input spike to *n*_1_ at frequency $f_{in}^{0}$. There exists a cutoff frequency $f_{c}^{i}$ for each neuron *n*_*i*_. If input spike frequency to *n*_*i*_ is lower than $f_{c}^{i}$, the neuron cannot spike. This value can be mapped to original input frequency $f_{in}^{0}$ with (10) of the memory pathway ending at *n*_*i*−1_: $f_{c}^{i}=\Phi n_{i-1}\left(f_{in}^{0}\right)$. The mapped cutoff frequency is referred to as $f_{mapped}^{i}$. Since a neuron must receive at lease one input spike to reach threshold, along memory pathway $f_{mapped}^{i}$ decreases, or holds the same. Therefore, the longest duration *T* that the memory pathway can hold information is $T={\left(f_{mapped}^{m}\right)}^{-1}$. Using (10), we have: $f_{c}^{m}=\Phi n_{m-1}\left(f_{mapped}^{m}\right)$, which leads to:

(14) $$\begin{array}{l} f_{mapped}^{m}={\left(\frac{1}{f_{c}^{m}}-r-r\overset{m-3}{\underset{k=0}{\sum }}\overset{k}{\underset{j=0}{\prod }}\gamma_{m-1-j}\right)}^{-1}\overset{m-1}{\underset{i=1}{\prod }}\gamma_{i} \end{array}$$

(15) $$\begin{array}{l} T=\left(\frac{1}{f_{c}^{m}}-r-r\overset{m-3}{\underset{k=0}{\sum }}\overset{k}{\underset{j=0}{\prod }}\gamma_{m-1-j}\right){\left(\overset{m-1}{\underset{i=1}{\prod }}\gamma_{i}\right)}^{-1} \end{array}$$

Following the same process used in proof of Lemma 2.2, we can show that *T* is different for each memory pathway. The proof is complete.

## 3. Results

### 3.1. Parameters and Simulation Configurations

In this work we use a GPU accelerated SNN simulator we developed as an open source project named ParallelSpikeSim (She et al., 2019). The manually tuned neuron parameters are: for learner neurons, *a* is –4.1, *b* is –0.01, *c* is 0.31, and *r* (refractory period) is 20 ms; for short-term neurons, *a* is –5.1, *b* is –0.02, *c* is 0.45, and *r* is 10 ms; for long-term neuron, *a* is –1.6, *b* is –0.001, *c* is 0.16, and *r* is 10 ms. Values of STDP parameters are: α_*p*_ = 0.1, α_*d*_ = 0.03, *G*_*max*_ = 1.0, *G*_*min*_ = 0, τ_*pot*_ = 10 ms, and τ_*dep*_ = 80 ms. To prevent early convergence of synaptic conductance and allow the network to effectively learn the entire dataset, the values of α_*p*_ and α_*d*_ are chosen to be relatively small, and training data is shuffled for both class and motion categories. In terms of simulation, the unit timestep is set to be 1 ms. Input frames are converted to spike trains with pixel intensity proportional to spike frequency range $f_{min}^{input}=\text{0}\;\text{Hz}$ and $f_{max}^{input}=\text{100}\;\text{Hz}$. Time spent on each frame is *t*_*train*_ = 300 ms. All neuron states are reset to default value after learning of each sequence.

### 3.2. Quantitive Analysis

Comparing *t*_*decay*_ when *m* is a long-term neuron and when *m* is a short-term neuron, starting from the same membrane potential level, the ratio of the time it takes for two neurons to decay to *v*_*reset*_ is:

$$\frac{t_{decay}^{ltn}}{t_{decay}^{stn}}\approx 2.4$$

From (13), we can find the cut-off frequency value by setting Δ*v*_*m*_ = 0, which gives:

$$\left(v_{reset}+\frac{a}{b}\right)\left(e^{b\Delta t}-1\right)=-cGe^{b\Delta t}$$

$$\Delta t=\frac{1}{b}ln\left(\frac{v_{reset}+a/b}{v_{reset}+a/b-cG}\right)$$

In terms of cut-off frequency,

$$f_{0}=\frac{1}{\Delta t}=\frac{b}{ln\left(\frac{v_{reset}+a/b}{v_{reset}+a/b-cG}\right)}$$

Comparing three neuron types with values of parameters {*a, b, c*} as shown before, under different synapse conductance, the cut-off frequency of each neuron is shown in Table 1. It can be observed that, for all three neuron types, the cut-off frequency has a strong dependency on synapse conductance.

**Table 1:** Cut-off frequency *f*_0_ (Hz) of post-synaptic neuron with one pre-synaptic neuron as input at different synapse conductance.

**Neuron type**	**G = 0.1**	**G = 0.2**	**G = 0.3**	**G = 0.4**	**G = 0.5**	**G = 0.6**
Learner	105.8	52.9	35.2	26.4	21.1	17.6
Short-term	78.9	39.5	26.3	19.7	15.8	13.1
Long-term	63.5	31.7	21.1	15.8	12.7	10.6

### 3.3. Baseline Networks

Five DNNs are implemented to represent baselines for spatiotemporal processing, namely, (i) a simple 3D CNN (Tran et al., 2015) referred to as 3D CNN-α, which has similar layer configurations as H-SNN, (ii) 3D CNN-β, a more complex 3D CNN with more layers and parameters, (iii) 3D MobileNetV2 and (iv) 3D ShuffleNetV2 as implemented in Köpüklü et al. (2019). The fifth baseline for comparison is an implementation of CNN+LSTM (Donahue et al., 2015). To prevent overfitting, for 3D CNN-α and 3D CNN-β dropout layers are applied; for all DNN baselines, early stopping for training are used. In Wu et al. (2018), spatiotemporal back-propagation is shown for SNN and the trained network is tested for dynamic dataset. We implement this design with two variants as additional bio-inspired baseline networks. The first variant is referred to as BP-SNN, which has the same convolution layer configuration as H-SNN but does not use neurons of different dynamics. Based on the original BP-SNN structure, we implement refractory period to the neurons, and modified each layer to include neurons with long and short memory, similar to H-SNN. This second variant is referred to as BP-SNN-LS.

### 3.4. Network Complexity and Energy Dissipation

The configurations of convolution layers and network parameter number are shown in Table 2. The tested H-SNN has 0.74 million parameters. 3D CNN-α has similar complexity as H-SNN with 0.83 million parameters and BP-SNN has the same number of parameters as H-SNN. On the other hand, 3D CNN-β and CNN+LSTM contains 4.5 million and 3.7 million parameters. 3D MobileNetV2 and 3D ShuffleNetV2 has 0.5x complexity (Köpüklü et al., 2019) and contains more parameters than H-SNN. For CNN+LSTM, parameters in the CNN encoder is 2.7M, while that in the LSTM decoder is 1.0M.

**Table 2:** Network configurations.

**Model**	**Convolution layer configuration**	**Total parameter**
3D CNN-α	Conv3D{[3x3x3,20],[3x3x3,32], [5x5x5,64], [5x5x5,64]}	0.83M
3D CNN-β	Conv3D{[3x3x3,32],[5x5x5,64], [3x3x3,96], [3x3x3,128]x2}	4.5M
3D MobileNetV2	(Köpüklü et al., 2019)	1.5M
3D ShuffleNetV2	(Köpüklü et al., 2019)	1.2M
CNN+LSTM	Conv2D{[3x3,64],[3x3,128],[5x5,256]}	3.7M
BP-SNN/BP-SNN-LS	Conv2D{[3x3,32],[3x3,64], [5x5,128], [7x7,40]}	0.74M
**H-SNN**	Conv2D{[3x3,32],[3x3,64], [5x5,128], [7x7,40]}	0.74M

For H-SNN, network memory consist of two main parts: (i) synapse conductance, which are trainable parameters, use 0.474M, and (ii) neuron state, which are non-trainable variables, use 0.267M to store all membrane potential. The number of H-SNN's trainable parameters is thus significantly less than 3D CNN-β and CNN+LSTM. Moreover, it is well-known that the event-driven nature of SNN assists in reducing network activation which in turn reduced energy dissipation of computation. Using method presented in Panda et al. (2020), we compute the energy advantage of H-SNN over DNN baselines during inference as follows: 1.0 for H-SNN, 1.55 for 3D CNN-α, and 3.37 for 3D ShuffleNetV2; 3D MobileNetV2, 3D CNN-β, and CNN+LSTM consumes 9.01×, 11.80× and 28.88× higher energy than H-SNN, respectively. BP-SNN and BP-SNN-LS uses similar energy as H-SNN.

### 3.5. Single-Objective Prediction

#### 3.5.1. Experimental Details

To test the effectiveness of H-SNN in learning spatiotemporal patterns, both single-objective and multi-objective experiments are conducted. In the single-objective experiment, an event camera dataset of human gesture (Amir et al., 2017) is used. Here, individual events are superimposed onto frames with resolution of 128x128 over 20 ms window. Each generated sequence contains one hundred frames and the network learns to predict the type of action in each sequence. Three baseline networks are tested: 3D CNN-α as well as the more complex 3D MobilNetV2 and 3D ShuffleNetV2.

To study the feasibility of learning with less labeled data and benefit of unsupervised learning, three sets of experiments are performed on DNN baselines using different training set sizes: 100, 50, and 30% of the full training data set. In terms of H-SNN, two training configurations are tested: one that uses the same training set as DNN for STDP learning and SGD-based final layer tuning, referred to as **H-SNN**; the other, referred to as **H-SNN (full data)**, uses the full training set for STDP unsupervised learning, while SGD-based tuning uses the same set as DNN.

#### 3.5.2. Results

As shown in Table 3, accuracy results from the three sets of experiments are listed. With full training dataset, accuracy of H-SNN is on a comparable level with 3D MobileNetV2 and 3D ShuffleNetV2 and outperforms 3D CNN-α. With less amount of labeled training data, all networks experience performance degradation. Among tested networks, H-SNN (full data) has the lowest accuracy decrease, while other networks lose around 7% from 100% training data to 30%. Such difference leads to the higher accuracy of H-SNN (full data) in low training data conditions, which indicates that unsupervised STDP learning of unlabeled data is effectively improving spatiotemporal pattern recognition in this particular task.

**Table 3:** Accuracy result of event camera dataset for networks trained with 100, 50, and 30% of labeled training data.

**Model**	**100%**	**50%**	**30%**
3D CNN-α	92.8	90.5	86.3
3D MobileNetV2	97.0	94.2	90.4
3D ShuffleNetV2	97.3	95.4	90.1
**H-SNN**	96.2	93.8	**90.9**
**H-SNN (full data)**	96.2	**95.8**	**93.7**

*H-SNN accuracy results that exceed all baselines are marked in bold*.

### 3.6. Multi-Objective Prediction

#### 3.6.1. Experimental Details

The second set of experiments is designed as a multi-objective computer vision task: the network observes a moving object as visual input to predict the class of the object and dynamic of its motion. We generate dataset with controlled motion dynamics by extracting objects from an aerial image dataset (Xia et al., 2017). A subset (20%) of the original training data for each object class is used with label to test the benefit of unsupervised learning. In order to generate transformation sequences, objects are placed on canvas and applied with translation and rotation, each with five possible dynamics: static, constant speed, accelerating, decelerating, and oscillating. For the training sequence, transformation dynamics are generated with parameters *P*_*train*_. For the test sequence, objects are taken from the test set of Xia et al. (2017) and transformation dynamic parameters are drawn from Gaussian distribution with mean *P*_*train*_ and standard deviation σ_*ts*_. As a second, non-aerial test case, we have performed similar experiments on Fashion-MNIST dataset which are 10 classes of apparel items with the dimension of 28 by 28, as objects. Training and test sequences are generated following the same process as discussed above, except that 10% of the original training data is used. For all generated sequences, training data is shuffled for both object class and motion dynamic.

In addition to regular training/inference setup, to understand the network's capability to learn class and motion independently, a total of three sets of experiments are conducted:

- ***Experiment 1: all-objective prediction*** In this regular training/inference setup, training set contains all classes and all possible transformation dynamics, and networks are tested for prediction of object class and its rotation/translation dynamics, which is either static, constant speed, accelerating, decelerating, or oscillating.
- ***Experiment 2: class-agnostic motion prediction*** In this experiment, networks are trained with sequences containing objects belonging to half of all classes, and tested for transformation dynamics prediction on the other half classes.
- ***Experiment 3: motion-agnostic class prediction*** Training sequences contain all object classes, but with only a subset of motion dynamics. Networks are tested for accuracy of class prediction but with unknown dynamics.

**Figure 5:**
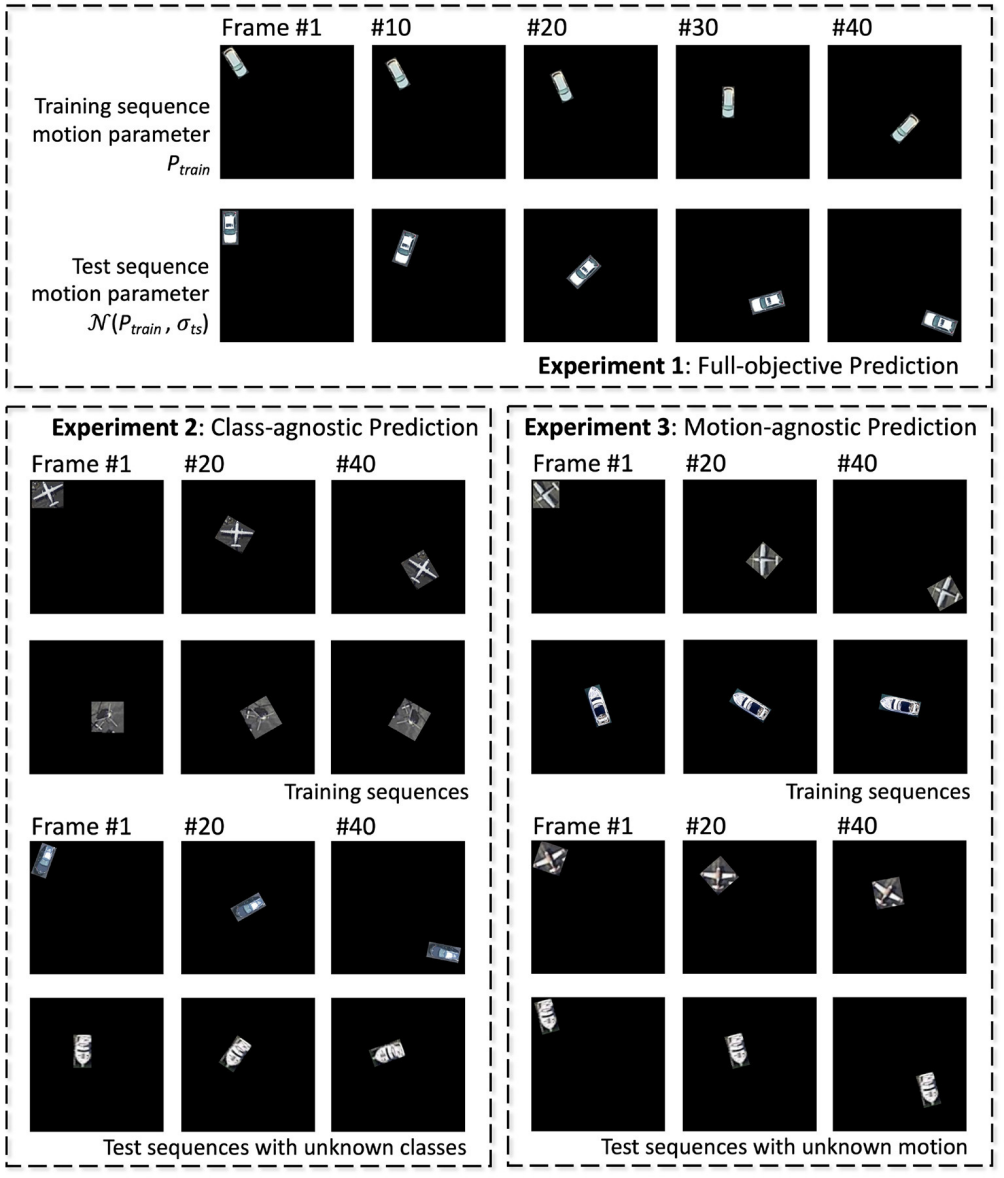
Illustrations of training and test sequences for the three experiments of the aerial footage dataset.

Experiment 1 is conducted on both the aerial and Fashion-MNIST dataset, while the other two are tested on the aerial dataset only. Examples of training/test sequences for the three experiments are shown in Figure 5. All baseline networks as discussed in section 3.3 are tested.

#### 3.6.2. Training Configurations

We test H-SNN with two training schemes for the aerial dataset. The first one, referred to as **H-SNN (1xU)**, uses 20% of the training set mentioned before for STDP learning and SGD-based final layer tuning. To study the advantage of training with unlabeled data, we create a second network, **H-SNN (5xU)**, that uses 5× more unlabeled data during STDP learning in SNN while the SGD-based tuning of the last year still uses the original labeled training set same as H-SNN (1xU). For Fashion-MNIST, **H-SNN (1xU)** uses sequences generated with 10% of original training set per class for unsupervised learning and final layer tuning; **H-SNN (10xU)** uses the whole training set for unsupervised learning and the same 600 objects per class sequences as used in H-SNN (1xU) for final layer supervised tuning. All baselines, including DNNs and SNN trained with back-propagation, are trained with the dataset used by H-SNN (1xU).

#### 3.6.3. Aerial Footage Results

##### 3.6.3.1. All-objective prediction

In all-objective prediction, results are measured by four metrics: accuracy for three separate targets and joint accuracy, which accounts for predictions that are correct for all three separate targets. Each objective's individual accuracy: class, rotation, and translation, is referred to as C, R, and T. Training accuracy for all networks are shown in Table 4. From the test accuracy results in Table 5, it can be observed that 3D MobileNetV2 and 3D ShuffleNetV2 show better accuracy than 3D CNN-α while they both fall behind 3D CNN-β and CNN+LSTM. BP-SNN-LS demonstrates better performance than BP-SNN in rotation and translation targets, while its class prediction is similar with BP-SNN.

**Table 4:** Training accuracy of all-objective prediction with aerial footage dataset.

**Model**	**Joint**	**{C,R,T}**
3D CNN-α	80.5	90.8, 94.9, 93.4
3D CNN-β	84.8	91.3, 96.5, 96.3
3D MobileNetV2	88.4	92.4, 97.7, 97.9
3D ShuffleNetV2	87.8	92.6, 96.8, 97.9
CNN+LSTM	86.7	90.7, 97.5, 98.1
BP-SNN	87.4	89.5, 98.9, 98.7
BP-SNN-LS	85.8	90.4, 97.5, 97.3
**H-SNN (1xU)**	84.8	91.4, 96.5, 96.1
**H-SNN (5xU)**	89.6	92.3, 98.1, 99.0

**Table 5:** Test accuracy of all-objective prediction with aerial footage dataset.

	**σ_*****ts*****_ = 1**		**σ_*****ts*****_ = 3**		**σ_*****ts*****_ = 5**	
**Model**	**Joint**	**{C,R,T}**	**Joint**	**{C,R,T}**	**Joint**	**{C,R,T}**
3D CNN-α	51.3	64.5, 87.9, 90.4	23.2	58.9, 61.9, 63.5	16.0	56.1, 51.1, 55.8
3D CNN-β	58.6	67.1, 94.7, 92.2	31.6	64.9, 66.1, 73.7	25.3	62.8, 60.3, 66.9
3D MobileNetV2	54.7	66.9, 88.3, 92.7	24.2	64.1, 53.8, 70.1	18.0	61.9, 47.1, 62.0
3D ShuffleNetV2	53.8	64.8, 89.8, 92.5	26.1	63.2, 55.8, 73.9	19.9	60.8, 49.7, 65.9
CNN+LSTM	56.1	66.2, 92.8, 91.3	28.4	63.0, 68.3, 66.0	23.3	61.3, 63.8, 59.5
BP-SNN	49.5	65.1, 86.7, 88.3	23.6	61.7, 59.2, 64.6	17.4	60.4, 50.1, 57.3
BP-SNN-LS	58.1	66.7, 92.6, 94.1	32.5	60.1, 68.8, 78.7	26.7	58.3, 65.3, 70.2
**H-SNN (1xU)**	56.2	66.8, 91.0, 92.4	**34.0**	64.4, **70.5**, 74.8	**29.0**	**63.2**, **66.1**, 69.7
**H-SNN (5xU)**	**68.0**	**72.8**, 94.4, **98.8**	**44.6**	**69.5**, **78.4**, **81.8**	**32.9**	**66.4**, **68.3**, **72.6**

With σ_*ts*_ = 1, H-SNN (1xU) predicts with good accuracy for motion dynamics and achieves a reasonable level of class prediction accuracy. This indicates that H-SNN is able to learn spatiotemporal patterns from moving objects and predicts for separate objectives based on the learned patterns. Comparing H-SNN (5xU) to H-SNN (1xU), unsupervised learning provides considerable performance increase for all targets. With increasing σ_*ts*_, accuracy for class prediction does not experience drastic degradation, showing that visual features learned by the network have a high degree of transformation invariance.

In comparison with baseline networks, accuracy values where H-SNN exceeds all baselines are marked bold in Table 5. For σ_*ts*_ = 1, H-SNN (1xU) outperforms BP-SNN and 3D CNN variants except for 3D CNN-β, while H-SNN (5xU) shows accuracy on a par with 3D CNN-β and CNN+LSTM. The advantage of H-SNN (1xU) is more evident in predicting motion with high deviation, as it achieves better results than baseline networks. This indicates that H-SNN is able to generalize more effectively the transformation invariant/equivariant patterns. With extra unlabeled dataset used for SNN learning, H-SNN (5xU) outperforms all baselines networks in most metrics. BP-SNN shows comparable accuracy as H-SNN (1xU) for class prediction, while its performance for motion prediction is noticeably lower than H-SNN (1xU), especially at higher σ_*ts*_. BP-SNN-LS has similar performance with H-SNN (1xU) while still outperformed by H-SNN (5xU).

**Figure 6:**
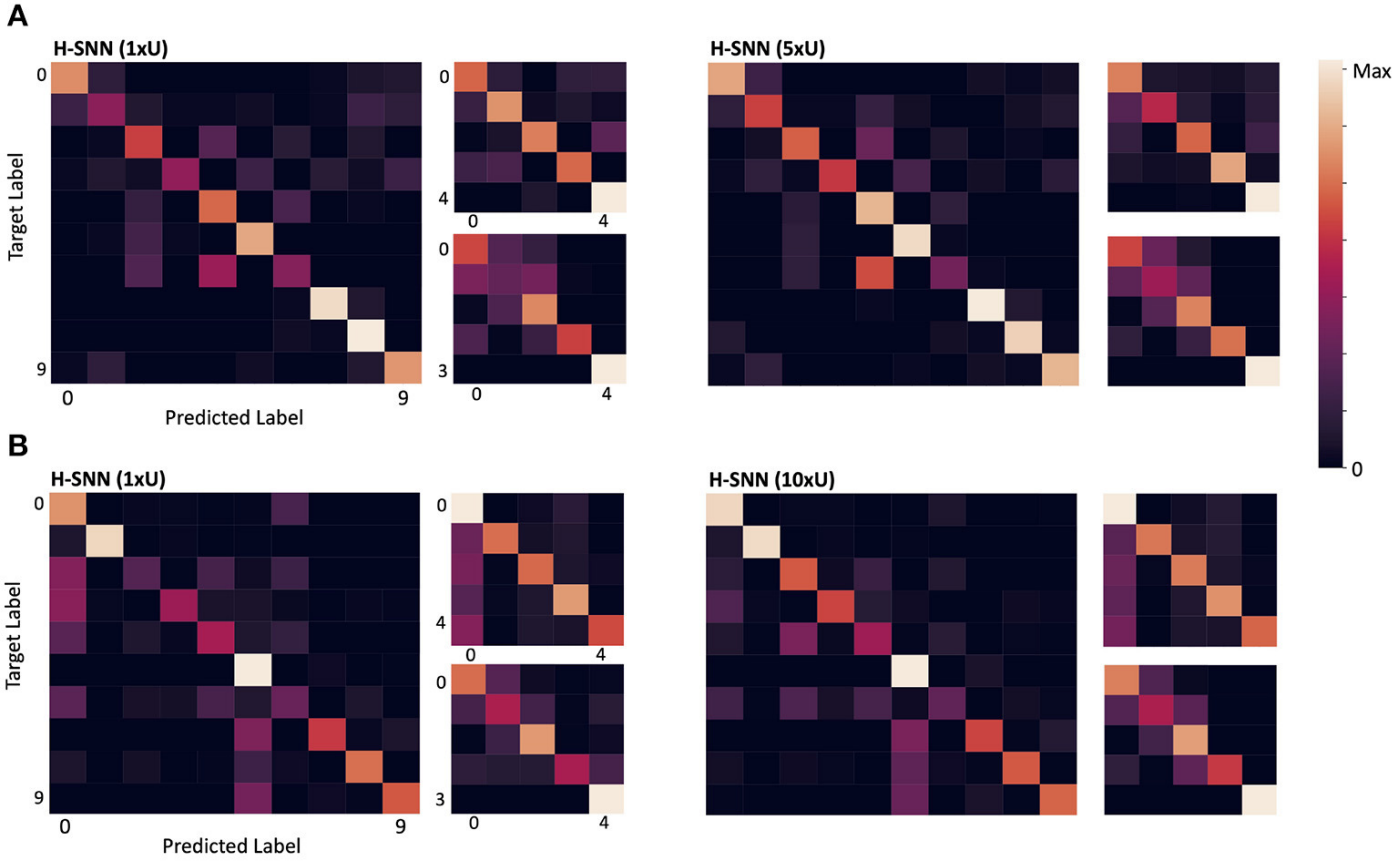
Confusion matrix for **(A)** aerial image sequences and **(B)** Fashion-MNIST sequences; for each network, the left matrix is for class prediction, the top right for rotation and the bottom right for translation; darker color represents less instances.

Confusion matrices for σ_*ts*_ = 5 are shown in Figure 6A. For each of the two variants of H-SNN, results are presented with three matrics. The matrix on the left is for class prediction, the top right is for rotation and the bottom right is for translation. Horizontal axis is predicted label and vertical axis is target label, each marked with a number; for class prediction, 0-9 represents the 10 classes of objects; for rotation and translation, 0 is static, 1 is constant speed, 2 is acceleration, 3 is deceleration, and 4 is oscillation. Lighter color represents more instances. It can be observed that, in terms of class prediction, confusion matrices of H-SNN (1xU) and H-SNN (5xU) share similarities, while H-SNN (5xU) predicts with more consistency across all classes. For motion prediction, learning unlabeled data has different effect: errors of H-SNN (5xU) for rotation prediction is more concentrated in one dynamic, while its errors for translation prediction spreads out more evenly, compared to H-SNN (1xU).

##### 3.6.3.2. Class-agnostic motion prediction

Table 6 lists accuracy of class-agnostic motion prediction. Each cell in the table contains accuracy for rotation (R) and translation (T). Result shows that the two H-SNN implementations are able to successfully predict motion dynamics of objects from unknown classes. Compared to motion dynamic accuracy from all-objective prediction, we observe that H-SNN experiences some degree of performance degradation. However, the decrease in accuracy is not drastic, especially for H-SNN (5xU). This shows that H-SNN is able to learn and predict motion dynamics independent of object's visual features on a certain level.

**Table 6:** {R,T} prediction accuracy for unknown classes with aerial footage dataset.

	****σ_*ts*_ = 1****	****σ_*ts*_ = 3****	****σ_*ts*_ = 5****
**Model**	**{R,T}**	**{R,T}**	**{R,T}**
3D CNN-α	86.4, 88.7	60.7, 64.9	52.3, 56.3
3D CNN-β	95.2, 91.4	70.3, 65.3	61.4, 59.3
3D MobileNetV2	91.0, 83.6	67.1, 67.4	56.0, 61.6
3D ShuffleNetV2	97.5, 81.9	68.2, 62.2	67.6, 57.8
CNN+LSTM	93.1, 87.6	72.5, 66.7	63.1, 57.7
BP-SNN	84.7, 85.9	57.5, 64.2	51.4, 58.6
BP-SNN-LS	86.5, 94.8	62.3, 78.2	57.5, 60.4
**H-SNN (1xU)**	88.6, 91.0	68.0, 73.4	64.0, **63.2**
**H-SNN (5xU)**	92.3, **98.4**	**72.7**, **80.7**	66.3, **70.7**

*H-SNN accuracy results that exceed all baselines are marked in bold*.

Among conventional deep networks, 3D CNN-β and CNN+LSTM show better accuracy than 3D CNN-α by a significant lead. 3D ShuffleNetV2 performs well in predicting rotation dynamic, while 3D MobileNetV2 shows good accuracy for translation dynamic. BP-SNN-LS has good performance for translation prediction, while the spatiotemporal patterns learned by BP-SNN are less generalizable, as its performance lags behind H-SNN and other baselines in this test. With increasing variation in transformation parameters, as observed in the σ_*ts*_ = 3 and σ_*ts*_ = 5 cases, accuracy of all networks degrade considerably. Accuracy of H-SNN (1xU) is on similar level with the more complex DNNs and BP-SNN-LS, and higher than 3D CNN-α and BP-SNN. H-SNN (5xU) shows comparable or better performance than best baseline performance. Similar to all-objective prediction, the advantage of H-SNN (5xU) is more evident for high σ_*ts*_ cases.

##### 3.6.3.3. Motion-agnostic class prediction

The three training/test pairs of motion dynamics are shown in Table 7 (left), and results for each pair is shown in Table 7 (left). H-SNN is able to learn motion invariant spatial patterns as it predicts object classes with reasonable accuracy. For this task, accuracy of H-SNN (1xU) can again be improved further using more unlabeled data as shown in the H-SNN (5xU) results. This indicates a better generalization ability of H-SNN (5xU) for objects with unknown transformations.

**Table 7:**
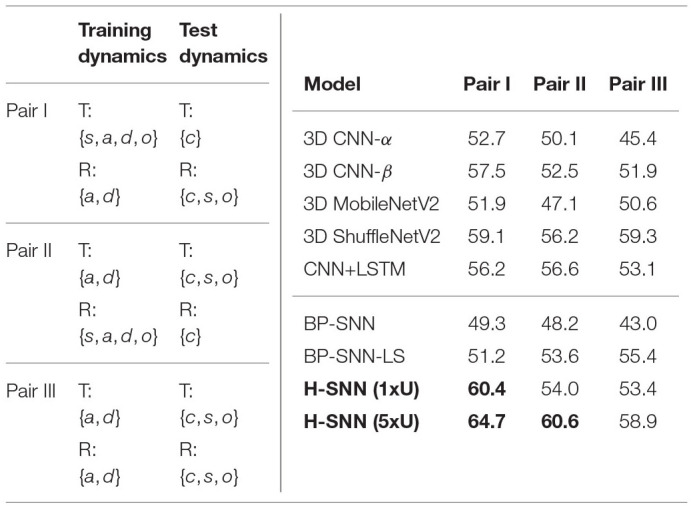
Motion-agnostic class prediction: (left) configurations for class prediction test with unknown transformations where {*s, c, a, d, o*}: static, constant speed, accelerating, decelerating and oscillating; (right) accuracy of class prediction for different test cases with aerial footage dataset.

*H-SNN accuracy results that exceed all baselines are marked in bold*.

In this test, 3D ShuffleNetV2 provides better performance than all other baselines. Compared to other networks, H-SNN (1xU) performs well for Pair I while keeping its advantage over 3D CNN-α and BP-SNN for all test cases. For Pair II, it is on a par with the best baseline results. H-SNN (5xU) achieves considerable improvement for all pairs by providing the best accuracy results except for Pair III, where it slightly falls behind 3D ShuffleNetV2. Hence, we observe that patterns learned from unlabeled data by STDP reduces the impact of unknown transformations to class prediction. For BP-SNN, degradation from all-objective prediction is significant, which indicates that the network has difficulty in learning spatial patterns invariant to unknown transformations. Compared to BP-SNN, BP-SNN-LS shows improvement in Pair II and Pair III while performs similarly in Pari I.

It is also worth noting that, compared to previously shown class prediction results, combinations of training/test dynamics affect network performance differently. For example, Pair II causes more degradation to 3D CNN-β than to CNN+LSTM while Pair I has similar influence to the two networks. For BP-SNN, Pair III shows to be most challenging. This indicates that the spatial patterns learned by networks generalize differently for unseen motion dynamics.

#### 3.6.4. Fashion-MNIST Results

As shown in Table 8, both variants of H-SNN provide good accuracy for the three targets, indicating that the network is able to effectively learn the spatiotemporal patterns in moving apparel items, which have different visual features from the aerial footage dataset. H-SNN (10xU) shows higher accuracy than H-SNN (1xU) for class and translation motion prediction, while the improvement it achieves for rotation prediction is smaller.

**Table 8:** Accuracy result for sequences generated with Fashion-MNIST.

	**σ_*****ts*****_ = 1**		**σ_*****ts*****_ = 3**		**σ_*****ts*****_ = 5**	
**Model**	**Joint**	**{C,R,T}**	**Joint**	**{C,R,T}**	**Joint**	**{C,R,T}**
3D CNN-α	54.3	70.4, 86.4, 89.2	19.0	50.2, 57.0, 66.5	10.9	43.9, 45.9, 54.4
3D CNN-β	63.1	72.5, 92.6, 94.0	28.4	61.0, 61.3, 75.9	18.2	58.9, 53.8, 57.4
3D MobileNetV2	57.6	70.0, 94.2, 87.3	22.6	58.3, 57.4, 67.6	13.8	53.8, 48.2, 53.1
3D ShuffleNetV2	61.0	68.7, 92.3, 96.2	23.9	53.4, 56.0, 79.8	12.1	43.2, 47.5, 59.0
CNN+LSTM	55.7	69.2, 89.6, 89.8	24.2	55.3, 74.7, 58.5	18.4	53.2, 64.1, 54.1
BP-SNN	54.5	68.1, 85.7, 93.4	19.5	47.0, 56.5, 73.5	14.3	50.6, 47.5, 59.3
BP-SNN-LS	70.0	76.8, 97.1, 94.1	37.2	66.0, 71.6, 78.7	26.0	62.5, 67.3, 61.7
**H-SNN (1xU)**	65.6	74.1, 96.2, 92.2	**38.9**	64.8, **83.7**, 71.8	**27.6**	59.2, **71.0**, **65.7**
**H-SNN (10xU)**	**73.9**	**79.4**, 96.8, **96.2**	**47.5**	**70.0**, **84.9**, **79.9**	**31.7**	**65.2**, **71.9**, **67.6**

*H-SNN accuracy results that exceed all baselines are marked in bold*.

From the confusion matrix in Figure 6B, the two H-SNN variants show similar profile for rotation and translation predictions while their class prediction differentiates. With unsupervised learning, H-SNN (10xU) is able to predict more accurately for classes that have high error rate, while the improvement on classes with lower error rate is less noticeable.

Among the baseline DNNs, 3D CNN-β shows good result at low σ_*ts*_ as it performs better than other deep networks and BP-SNN, and shares similar performance with CNN+LSTM in high σ_*ts*_ in terms of joint accuracy while each individual target differs. BP-SNN-LS shows considerable gain from BP-SNN and has the best performance among baseline networks. H-SNN (1xU) demonstrates similar accuracy as 3D CNN-β for σ_*ts*_ = 1 while its accuracy is lower than BP-SNN-LS. At higher σ_*ts*_, H-SNN (1xU) shows comparative advantage: the prediction accuracy for multiple targets exceeds all baseline networks. This indicates that, for Fashion-MNIST, H-SNN is able to more effectively learn spatiotemporal patterns generalizable to different transformation dynamics. With unsupervised learning of extra unlabeled sequences, H-SNN (10xU) is able to make better prediction in almost all individual targets, and achieves joint accuracy considerably higher than baseline networks.

#### 3.6.5. Impact of Training Data Size

In this section we investigate the impact of scaling labeled training data on H-SNN, with two types of unsupervised learning setups. For the aerial dataset, unsupervised learning and supervised training of H-SNN both use sequences generated with 20, 60, and 100% of the original dataset. For Fashion-MNIST, three levels of supervised training: 10, 50, and 100%, are tested on a network that learns 100% data without supervision. The results are shown in Table 9. For the aerial dataset, it can be observed that by increasing training data size the network experiences considerable improvement on performance. The gain in class prediction accuracy is higher than that in motion prediction and the improvement in general is higher for lower σ_*ts*_ cases. When the network always learns 100% unlabeled data, similar trend can also be observed as shown in the Fashion-MNIST result. However, the benefit from increasing training data size is smaller than in the aerial dataset, e.g., for σ_*ts*_ = 1, joint accuracy increased by around 27% for aerial image, while for Fashion-MNIST the gain is around 9%.

**Table 9:** Impact of training data size for aerial dataset (top 3 rows) with scaling unsupervised learning data size and Fashion-MNIST (bottom 3 rows) with fixed unsupervised learning data size.

	**σ_*****ts*****_ = 1**		**σ_*****ts*****_ = 3**		**σ_*****ts*****_ = 5**	
**Training set**	**Joint**	**{C,R,T}**	**Joint**	**{C,R,T}**	**Joint**	**{C,R,T}**
20%	56.2	66.8, 91.0, 92.4	34.0	64.4, 70.5, 74.8	29.0	63.2, 66.1, 69.7
60%	76.5	81.5, 95.2, 98.6	49.0	73.7, 81.7, 81.3	34.6	70.4, 69.0, 71.2
100%	83.5	86.3, 97.2, 99.5	54.7	77.1, 84.9, 83.5	37.8	72.9, 70.5, 73.5
10%	73.9	79.4, 96.8, 96.2	47.5	70.0, 84.9, 79.9	31.7	65.2, 71.9, 67.6
50%	77.8	81.1, 97.6, 98.3	51.5	72.3, 86.5, 82.3	34.7	68.7, 73.6, 68.6
100%	82.3	85.3, 97.9, 98.6	57.4	76.8, 87.1, 85.9	38.1	74.2, 74.0, 69.4

## 4. Discussion

In this paper we present H-SNN as a novel spiking neural network design that is capable of learning spatiotemporal information with STDP. For H-SNN, no recurrent connection is needed due to the hierarchical formation of long and short memory. This makes it possible to implement a feedforward convolutional network that can be learned with STDP unsupervised training. The effectiveness of H-SNN design is confirmed through mathematical analysis and experiments.

Based on neuron design and network architecture, analysis of neuron dynamics is performed and formula of memory pathway response function is derived. We demonstrate that distinct response functions for different input spike frequency are present in H-SNN. Meanwhile, derivation of cut-off frequency of memory pathway shows that memory of different time scales can be achieved. H-SNN is thus analytically shown to be able to represent distinguishable temporal patterns.

We test H-SNN in computer vision tasks predicting for both single and multiple objectives, and demonstrate the effectiveness of H-SNN on dataset with different visual features and varying motion dynamics. H-SNN is compared with conventional DNN approaches including multiple variants of 3D-CNN, CNN with recurrent connections and SNN trained with back-propagation. Results show two main advantages of H-SNN. First, H-SNN has comparable accuracy with DNN using the same amount of training data. Meanwhile, with the addition of unlabeled data, H-SNN can be further optimized with unsupervised STDP learning and provides higher accuracy than conventional DNN and BP-SNN. The advantage over baselines is most significant when motion dynamic has high deviation from training set. This trend is observed for H-SNN with and without extra unlabeled data to learn. The second advantage is that, with unsupervised learning, H-SNN demonstrates better generalization ability to unknown motion or classes in motion-agnostic and class-agnostic tests. Compared to BP-SNN, H-SNN more effectively learns transformation invariant spatial patterns as well as the general spatiotemporal patterns in the dataset. The combination of long and short term neurons in BP-SNN-LS produces considerable improvement over the original BP-SNN in terms of motion dynamics prediction accuracy. However, the supervised training method of BP-SNN-LS does not have the ability to learn unlabeled data, thus cannot benefit from the same technique used for H-SNN (5xU) and H-SNN (10xU) to further improve SNN performance.

In addition to prediction accuracy, the improved performance of H-SNN is achieved with much lower network complexity than conventional deep networks, and can be implemented in hardware with higher energy-efficiency. In conclusion, H-SNN provides an appealing solution for learning spatiotemporal patterns encountered in computer vision applications that have limited training data and/or constrained computing resources.

## Data Availability Statement

Publicly available datasets are used in this study. This data can be found here: https://captain-whu.github.io/DOTA/dataset.html, https://github.com/zalandoresearch/fashion-mnist.

## Author Contributions

XS developed the main concepts, performed simulation, and wrote the paper. All authors assisted in developing the concept and writing the paper.

## Conflict of Interest

The authors declare that the research was conducted in the absence of any commercial or financial relationships that could be construed as a potential conflict of interest.
